# Magnetic Field Measurements Based on Terfenol Coated Photonic Crystal Fibers

**DOI:** 10.3390/s111211103

**Published:** 2011-11-28

**Authors:** Sully M. M. Quintero, Cicero Martelli, Arthur M. B. Braga, Luiz C. G. Valente, Carla C. Kato

**Affiliations:** 1 Pontifical Catholic University of Rio de Janeiro, Rua Marquês de São Vicente 225, Rio de Janeiro, RJ 22453-900, Brazil; E-Mails: fc.abraga@puc-rio.br (A.M.B.B); luizguedes@puc-rio.br (L.C.G.V.); cckato@puc-rio.br (C.C.K.); 2 Department of Electronics, Federal University of Technology-Parana, Av Monteiro Lobato, s/n–km 04-Ponta Grossa, PR 84016-210, Brazil; E-Mail: cmartelli@utfpr.edu.br

**Keywords:** photonic crystal fiber, high birefringence, magnetic field sensing, air-silica structured fiber, microstructured fiber

## Abstract

A magnetic field sensor based on the integration of a high birefringence photonic crystal fiber and a composite material made of Terfenol particles and an epoxy resin is proposed. An in-fiber modal interferometer is assembled by evenly exciting both eigenemodes of the HiBi fiber. Changes in the cavity length as well as the effective refractive index are induced by exposing the sensor head to magnetic fields. The magnetic field sensor has a sensitivity of 0.006 (nm/mT) over a range from 0 to 300 mT with a resolution about ±1 mT. A fiber Bragg grating magnetic field sensor is also fabricated and employed to characterize the response of Terfenol composite to the magnetic field.

## 1. Introduction

Optical fiber magnetic field sensors appear as an important alternative to electrical sensors, such as Hall Effect based sensors, for monitoring magnetic field in applications such as power plants, where electric insulation and electromagnetic interference are an issue. They can be compact, mechanically robust and easily installed in remote locations. Given that optical fibers are conventionally made of silica, which is naturally a very good insulator, it is not difficult to find designs that make them immune to electromagnetic interference [1]. Examples of optical fiber sensors monitoring temperature, vibration, strain as well as electric current and magnetic field in the electric power system are found in the literature [2] and some products are starting to be commercialized, demonstrating that the technology is gaining maturity [3].

The Faraday effect is normally used as the basis for detecting and monitoring electric current using optical fibers. It relies on the light polarization rotation as function of the applied field and the amount of rotation is given by *β* = *νBd*, where *β* is the angle of rotation, *ν* is the Verdet`s constant, *B* is the magnetic flux and *d* the interaction length between the magnetic field and light. Although Faraday effect based sensors respond to magnetic fields, the needed length of fiber and the typical fiber and magnetic field geometry [3,4] make them unsuitable for localized magnetic field measurements. The optical resonance in diffractive gratings can be used to enhance the Faraday effect in silica fibers reducing the length of the sensing fibers [5]

An obvious way of relaxing the need for optical fiber sensors with long interaction lengths is the possibility of using fiber materials with a higher Verdet′s constant, such as some soft glasses [6,7]. These glasses, however, are typically mechanically fragile and do not withstand the temperature levels required in many industrial applications. An alternative approach relies on the integration of Terfenol particles into the optical fibers providing them with high magnetostriction properties. This possibility was investigated and demonstrated using fiber Bragg gratings which led to the development of localized magnetic field sensors [8–11]. Here a novel sensor concept based on an in-fiber interferometer is proposed using the modal interference of two polarization states within a high birefringence photonic crystal fiber. The use of a pure silica high birefringence photonic crystal fiber (HiBi PCF) fiber brings some advantages over standard doped core fibers such as long term and temperature stability and hydrogen resistance [12]. This means that a pure silica optical fiber sensor can, in theory, be intrinsically more robust to outdoor deployment than germanium doped core fiber sensors.

The proposed sensor is based on a modal interferometer assembled by evenly exciting the two orthogonally polarized eigenemodes of a high birefringence photonic crystal fiber. As the modes propagate down the HiBi fiber with different propagation constants, the relative phase changes and gives rise to a periodic interference pattern over a broadband spectrum. This spectrum depends upon the cavity length and effective refractive index, changes in either can be determined via the optical spectrum. Terfenol particles are coated around the fiber interferometer using an epoxy resin forming a composite material. In the presence of a magnetic field the magnetostrictive composite material deforms and induces strain on the optical fiber changing both the length and the refractive index of the fiber and consequently varying the interferometer output, *i.e.*, the broadband interference spectrum. Shifts in the interference pattern can hence be related to the applied magnetic field.

In the work presented here the sensor dependence with the magnetic field is studied experimentally. First, the composite material sensitivity to the applied magnetic field is determined by using an optical fiber Bragg grating, detailed information about this sensor can be found in [13]. This result is subsequently used to understand how the mechanical forces, originated from the magnetostrictive effect, act on the photonic crystal fiber sensor. Finally, a mechanical model illustrating the main force components present in the sensor head as a result of the applied magnetic field is proposed and discussed.

## 2. Magnetostrictive Composite Material

Resin hardness, volume fraction and particle size of Terfenol-D directly impact the response of the magnetostrictive composites. In a previous paper published by the authors the optimal composition for a magnetostrictive composite using Terfenol particles and a cycloaliphatic epoxy resin (AeroMarine 300/21) was determined. Detailed information on the fabrication procedure as well as the study of performance defining the best composition of the magnetic field sensitive material can be found in [13]. The best performance, *i.e.*, sensitivity, was achieved by using Terfenol particles with sizes on the order of 250 μm diluted in an epoxy resin with a 30% volume fraction of Terfenol-D. This composition was used to develop the sensor presented here.

The sensor head is accomplished by embedding the optical fiber within the magnetostrictive composite. The operating principle of the sensor relies on the magnetostrictive properties of Terfenol-D. When it is exposed to a magnetic field this material changes size, converting the magnetic energy into mechanical energy, *i.e.*, strain. The strain induced in the magnetostrictive composite by the magnetic field is subsequently transferred to the optical fiber. Hence, two of the most important sought characteristics for the composite material were, first, high adherence to glass and, secondly, the capacity to transfer to the fiber the majority of the strain generated within the magnetostrictive composite as a result of the particles alignment and deformation with the applied magnetic field. Fiber Bragg gratings were used as means of characterizing the response of the composite with the magnetic field by determining the longitudinal component of the strain.

## 3. HiBi PCF Fiber Magnetic Field Sensor

The proposed photonic crystal fiber sensor is based on a modal interferometer. This type of interferometers have been used to measure many physical quantities [14] and rely on the phase difference between the two orthogonally polarized fiber modes along the optical path, generating fringes over a broadband propagation spectrum. The optical phase difference (*ϕ*) induced by the PCF fiber at each polarization mode can be expressed as:

(1) $$\phi_{\mathrm{PCF}}=\frac{4\pi \;\mathrm{LB}}{\lambda }$$

where *B* is the phase modal birefringence, *L* is the optical path length within the PCF, and *λ* is the free-space wavelength. The phase dependence with wavelength and magnetic field for the resulting sensor is given by:

(2) $$\phi_{H}\;(\lambda ,\;H)=\frac{4\pi \;L_{H}(H)\;B_{H}(\lambda ,H)}{\lambda }$$

where *B_H_* and *L_H_* are the phase modal birrefringence and the optical path length within the PCF fiber as a function of *H*, respectively. The phase change Δ*ϕ(H)* for this interferometer as a function of a magnetic field variation applied along the fiber axis, can be re-written as:

(3) $${\Delta \phi }_{H}=\frac{{\partial \phi }_{H}}{\partial \lambda }\Delta \lambda +\frac{{\partial \phi }_{H}}{\partial H}\Delta H$$

By substituting Equation (2) into Equation (3) and differentiating it with respect to *λ* and *H* one gets:

(4) $${\Delta \phi }_{H}=-\frac{4\pi L_{H}}{\lambda^{2}}\left(B-\lambda \frac{{\partial B}_{H}}{\partial \lambda }\right)\Delta \lambda +\frac{4\pi L_{H}}{\lambda }\frac{{\partial B}_{H}}{\partial H}\Delta H+\frac{4\pi B_{H}}{\lambda }\frac{{\partial L}_{H}}{\partial H}\Delta H$$

where $\left(B-\lambda \frac{\partial B}{\partial \lambda }\right)$ is the well-know group modal birefringence (G) [12], hence:

(5) $${\Delta \phi }_{H}=-\frac{4\pi L_{H}G_{H}}{\lambda^{2}}\Delta \lambda +\frac{4\pi L_{H}}{\lambda }\frac{{\partial B}_{H}}{\partial H}\Delta H+\frac{4\pi B}{\lambda }\frac{{\partial L}_{H}}{\partial H}\Delta H$$

The second and third terms in the Equation (5) represent the components of loads, induced by the magnectostrictive effect acting on the optical fiber when the composite material is subjected to an external magnetic field, one along the fiber and another transversal to the fiber axis. The first term determines the phase shift dependence with wavelength.

The interferometric sensor probes a magnetic field by measuring the wavelength shift (Δ*λ*) of one interference valley of the broadband optical spectrum at different magnetic field. At each dip the optical modes are in phase (Δ*ϕ_H_* = 0) and, consequently, the Equation (5) can be re-arranged as:

(6) $$\frac{\Delta \lambda }{\Delta H}=\frac{\lambda B_{H}}{G_{H}}\frac{{\partial L}_{H}}{L_{H}\partial H}+\frac{\lambda }{G_{H}}\frac{{\partial B}_{H}}{\partial H}$$

Equation (6) is generic and governs the relationship between the wavelength shift (Δ*λ*) and the magnetic field changes (Δ*H*) for the developed of high birefringence optical fiber magnetic field sensor. An important feature is that the length of the fiber interferometer does not affect the sensitivity of the sensor Δ*λ*/Δ*H*, however, it does impact the resolution of the measurement. This happens because the longer the optical fiber, the larger the number of fringes, therefore, the higher the resolution in the determination of Δ*λ*.

**Figure 1.**
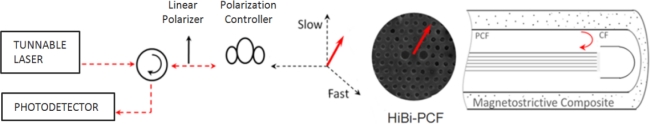
Schematic representation of the interferometric HiBi photonic crystal fiber sensor.

Figure 1 shows a schematic representation of the high birefringence photonic crystal fiber magnetic field sensor. The modal interferometer is characterized using a commercially available optical interrogator designed to read the reflection spectrum of optical fiber Bragg gratings (Micron Optics sm125). The sm125 Optical Sensing Interrogator employs full spectral scanning and data acquisition, providing measurements with high accuracy (1 pm).

**Figure 2.**
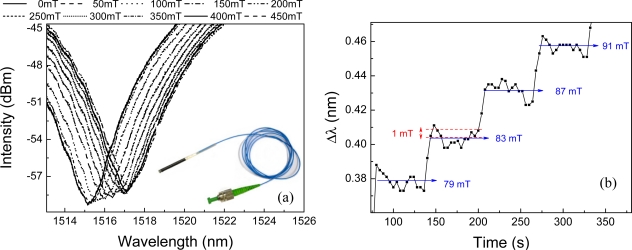
(**a**) Interferogram measured by the optical interrogator. Inset shows photo of the fabricated sensor head; (**b**) Magnetic field sensor resolution.

The light is launched into a single mode standard fiber (SMF28) which is connected to an in-fiber polarizer and a fiber polarization controller. The latter is used to align the light polarization at 45 degree with the HiBi PCF fiber axis, exciting both polarization states with approximately the same amount of power. One end of the 50 mm long PCF fiber is spliced to the polarization controller output whilst the other end receives an end cap (CF in Figure 1) to protect the air silica interface and keep its reflectivity constant [15]. The end cap is made of a capillary fiber with a hollow core of 50 μm in diameter and has one end gently spliced to the PCF fiber in order to avoid closing all the PCF fiber holes, the other end is collapsed by a fusion splicer. The splices were carried out with a conventional fusion splicer (Fujikura FSM-40S) operated in the manual mode. The back reflected signal is detected by a photo-detector and presents fringes or dips, such as the ones showed in Figure 2(a).

The number of fringes, as well as the distance between consecutive fringes, depend on the cavity length and on the effective refractive index along the light path. Since the interferometer operates in reflection the total cavity length is equivalent to 100 mm. Figure 2(a) shows the interference fringes of the sensor for different applied magnetic fields. The optical spectrum shifts towards longer wavelengths as the magnetic field is increased. A photo of the manufactured sensor head is shown in the inset of Figure 2(a). Figure 2(b) illustrates the system response for small steps variation of the magnetic field maintained constant for approximately one minute in successively periods of time. The magnetic field resolution is about ±1 mT in our current implementation. It is equivalent to an interrogation resolution 6 pm.

## 3. Results and Discussion

**Figure 3.**
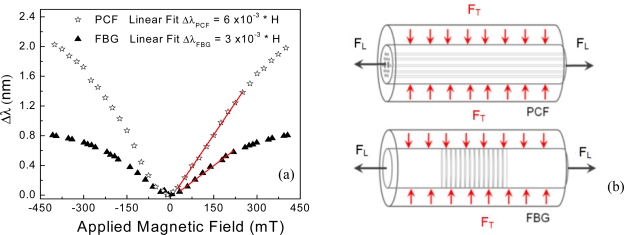
(**a**) Response of the two optical fiber sensors to magnetic field, white stars is the HiBi PCF fiber modal interferometer and black triangles the fiber Bragg grating; (**b**) Schematic representation of the forces acting on the optical fiber sensors. Black arrows indicate the elongation strain and the red arrows the resulting transversal compression forces induced both as a result of the magnetostrictive composite elongation for solid fiber with a Bragg grating and photonic crystal fiber.

The response of the interferometric sensor and the fiber Bragg grating reference sensor against the magnetic field is plotted in Figure 3(a) for comparison. Whilst the curves present approximately the same trend, it is possible to observe that the magnetic field has larger effect on the HiBi PCF fiber sensor. Both curves present a linear region which is highlighted by fitting linear curves and plotting them in red (Figure 3(a)). The angular coefficient for the HiBi PCF fiber modal interferometer is of about 0.006 (nm/mT) and the FBG 0.003 (nm/mT). The linear region for the PCF fiber sensor is also larger, ranging from 0 to 300 mT and the FBG’s from 0 to 250 mT.

It is relevant to point out that the sensors are not only based on different operating principles, but they also use different optical fibers. The Bragg grating is written in a solid circularly symmetric fiber which is little affected by hydrostatic forces. On the other hand, the interferometer uses a high birefringence fiber with holes whose sensitivity to hydrostatic forces is significant and well determined [16]. Nevertheless, using the result obtained with the FBG allows one to picture the way the mechanical forces induced by magnetostrictive composite affect the PCF. The overall net force can be divided into two main components acting on the optical fiber when the composite material is subjected to an external magnetic field, one longitudinal force along the fiber, represented by a tensile force (F_L_) (elongation), and another transversal to the fiber axis, represented by a compressive force (F_T_) (pressure).

Figure 3(b) shows a schematic representation of these forces acting on the optical fibers. The transversal force component arises due to the mismatch between the elastic properties of the magnetostrictive composite and the optical fibers by the Poisson’s effect. This is understandable considering the Poisson’s ratio of silica and Terfenol-D which are 0.17 and 0.3, respectively. Additionally, this component has more effect on the photonic crystal fiber, resulting in an increase of the sensitivity of the photonic crystal fiber in relation to the solid fiber optic with a Bragg grating. The effect of this transversal component on the sensor sensitivity can be represented by the second term of Equation (6) while the first one is responsible for the longitudinal component.

**Figure 4.**
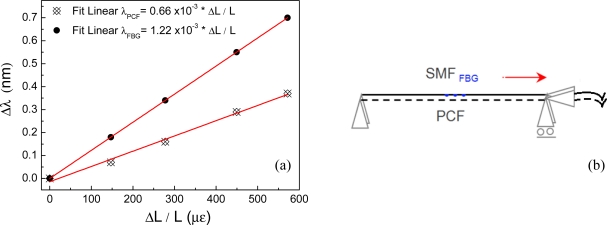
(**a**) Response of the HiBi PCF fiber modal interferometer and FBG to longitudinal strain; (**b**) Schematic of the experimental setup.

In order to try to understand how important these forces are in determining the sensitivity of the sensor another experiment was carried out to evaluate the optical sensors dependence with longitudinal strain. Figure 4 shows the response of the HiBi PCF modal interferometer and of a standard optical fiber Bragg grating to elongation strain. None of the fibers were embedded in the magnetostrictive composite. It is possible to observe that the Bragg grating presented a sensitivity to strain nearly twice as large as the HiBi PCF interferometer’s, ∂*λ*_FBG_/∂ɛ = 1.22 × 10^−3^ nm/μs and ∂*λ*_PCF_/∂ɛ = 0.66 × 10^−3^ nm/μs, respectively. This result is the opposite of that observed for the magnetic field sensors indicating that the compressive forces induced by the magnetostriction phenomenon at the Terfenol particles have an important contribution in sensitizing the holey optical fibers. Hence, the compressive stress component is responsible for a large contribution in increasing the performance of the PCF fiber sensor by inducing refractive index changes within the fiber and therefore maximizing the effect of the magnetic field over the optical fiber sensor. In this case, the second term of Equation (6) dominates representing the effect of the compressive force.

## 4. Conclusions

A novel optical fiber sensor obtained by integrating a HiBi PCF fiber with a custom made Terfenol-epoxy resin composite material is proposed. A simple mechanical model was used to explain the sensitivity as well as to assist the development of future magnetic field sensors using HiBi PCF fiber interferometers.

The manufactured sensor head was rather small when compared to other types of optical fiber magnetic field sensors based on the polarization rotation as a result of the Faraday effect. This gives rise to many possibilities in sensing of the magnetic fields within electrical equipment such as generators and motors. The use of the photonic crystal fiber provide characteristics which can be further explored in order to produce smaller and more sensitive sensors.

A new level of sensitivity and compactness could be achieved by using Bragg gratings written in photonic crystal fibers. The multiple pass properties allowed by resonating devices enhance the sensitivity to both RI and structural changes. Bragg gratings have been successfully written in air-silica structured fibers using special lasers [17–19]. This fabrication process has the down side, however, of increasing the amount of work as well as the cost of the final device.
